# Catalytic Performance of Palladium Supported on Sheaf-Like Ceria in the Lean Methane Combustion

**DOI:** 10.3390/nano10010031

**Published:** 2019-12-21

**Authors:** Shuna Li, Yagang Zhang, Jing Shi, Gang Zhu, Yanxiang Xie, Zhikai Li, Ruiyi Wang, Huaqing Zhu

**Affiliations:** 1 Xi’an Key Laboratory on Intelligent Additive Manufacturing Technologies, The Key Laboratory for Surface Engineering and Remanufacturing in Shaanxi Province, School of Chemical Engineering, Xi’an University, Xi’an 710065, China; zg_503a@163.com (G.Z.); yxxie@163.com (Y.X.); 2 Department of Chemistry and Chemical Engineering, Xi’an University of Science and Technology, Xi’an 710054, China; zhangyg04@126.com; 3 Department of Mechanical and Materials Engineering, University of Cincinnati, Cincinnati, OH 45221, USA; 4 State Key Laboratory of Coal Conversion, Institute of Coal Chemistry, Chinese Academy of Sciences, P.O. Box 165, Taiyuan 030001, China; lizhikai@sxicc.ac.cn (Z.L.); wangruiyi@sxicc.ac.cn (R.W.)

**Keywords:** lean methane combustion, sheaf-like, palladium, ceria

## Abstract

Sheaf-like CeO_2_ (CeO_2_-S) in microscale was prepared by the hydrothermal method, and then etched with KOH aiming to obtain an imperfect fluorite structure (CeO_2_-SK) with high content of oxygen vacancies and oxygen mobility. With CeO_2_-S and CeO_2_-SK as supports respectively, a modified colloidal deposition method was employed to obtain Pd/CeO_2_ catalysts for being used in lean methane combustion. According to the inductively coupled plasma (ICP), N_2_ physisorption and scanning electron microscopy (SEM) results, the Pd supported catalysts are very similar in their Pd loading, surface area and morphologies. SEM and transmission electron microscopy (TEM) results revealed various nanorods exposed CeO_2_ (110) and (100) facets on Pd/CeO_2_-SK surface after KOH etching. Raman spectra and H_2_-temperature programmed reduction (H_2_-TPR) results indicated that Pd/CeO_2_-SK catalyst has a much higher content of catalytic active PdO species than Pd/CeO_2_-S catalyst. It was also found that the catalytic performance of Pd/CeO_2_ in lean methane combustion depends greatly upon the exposing crystal planes and oxygen vacancies content of sheaf-like CeO_2_, and Pd/CeO_2_-SK exhibits higher activity than Pd/CeO_2_-S. The larger amount of CeO_2_ (110) and (100) planes on Pd/CeO_2_-SK surface can enhance the formation of oxygen vacancies, active Pd species and migration of lattice oxygen, which all evidently improve the redox ability and catalytic activity of the Pd/CeO_2_-SK catalysts in lean methane combustion.

## 1. Introduction

Currently, a great amount of coal mine methane needs to be utilized owing to the huge annual output of coal in the world [1,2]. The high concentration methane can be recovered into chemical feedstock; however, sometimes low concentration methane is discharged directly into the atmosphere without any treatment, which not only causes energy waste, but also brings serious environmental problems due to the global warming potential of CH_4_ is about 21–26 times higher compared with CO_2_ [3,4,5]. Catalytic combustion of methane is viewed as an effective measure to treat lean methane due to its high combustion efficiency and low emission of toxic products, such as CO and NO_x_ [6,7]. It is well known that the inertia of methane makes it difficult to oxidize at low temperatures. Thus, the design and preparation of catalysts with high activity in lean CH_4_ combustion at low temperatures still face great challenge.

Among the various catalysts, Pd supported catalysts are considered as one of the most promising candidates for methane combustion and activation owing to their extraordinarily high catalytic performance at low temperatures [7,8,9,10,11]. Furthermore, literature research has revealed that the rate limited step in methane combustion is the initial scission of the C–H bond [12], and palladium has the ability to split C–H bond [13]. According to Farrauto et al. the catalytic performance of palladium catalyst depends, to a great extent, on the nature of the support and the palladium-support interactions [14]. Thus, various oxide materials have been selected as carriers to prepare palladium supported catalysts for lean methane combustion.

Recently, ceria has attracted considerable attentions owing to its high oxygen storage capacity and remarkable redox properties [15,16,17,18], which permits effective oxygen adsorption and activation for CH_4_ oxidation. Hence, many Pd/CeO_2_ catalysts with exceptionally high activities for CH_4_ combustion at low temperatures have been synthesized and investigated in that the synergistic interaction between palladium and CeO_2_ may result in higher catalytic performance of Pd species [19,20,21]. Meanwhile, several reports have shown that the CeO_2_ morphology also has great influence on the catalytic activity of palladium loaded catalysts. This may be due to the fact that different shapes of ceria supports usually expose distinct ceria facets, which ultimately affects the palladium–CeO_2_ interaction. Tan et al. [22] found that Pd loaded on {110}-faced CeO_2_ nanocubes exhibited much higher activity in formaldehyde oxidation compared with Pd loaded on {111}-faced CeO_2_ nanooctahedrals and CeO_2_ nanorods exposed {100} and {110} planes. Lei et al. [23] indicated that Pd supported on octahedral CeO_2_ were much more active than those supported on rod and cube CeO_2_ in lean CH_4_ combustion owing to the synergistic effects between palladium species and (111) planes of octahedral CeO_2_ exposing. On the contrary, Guo et al. [9] believed that the larger activity of Pd/CeO_2_-microspheres in lean CH_4_ combustion was related to the exposed active (110) and (100) planes and its short porous channel. Up to now, various Pd/CeO_2_ catalysts with different shapes have been synthesized [9,22,23,24,25]; however, as far as we know, the preparation of ceria with sheaf-like morphologies in microscale and the shape effects of microscale ceria carrier on the catalytic activity of Pd/CeO_2_ catalyst in lean CH_4_ combustion have been rarely reported.

Therefore, in this work, sheaf-like CeO_2_ (CeO_2_-S) in microscale was synthesized by using the hydrothermal method. The as-made sheaf-like CeO_2_ was further etched with KOH to obtain an imperfect fluorite structure (CeO_2_-SK) with high content of oxygen vacancies as well as oxygen mobility, which can improve the activity of supported Pd catalysts in lean CH_4_ combustion at low temperatures. With CeO_2_-S and CeO_2_-SK as support respectively, Pd/CeO_2_ catalysts were synthesized by a modified colloidal deposition method as described previously with the aim to obtain a deep insight into the effect of the CeO_2_ support structure on the performance of Pd/CeO_2_ catalysts in lean CH_4_ combustion [16,26,27]. The relationship between the crystal planes exposing and oxygen vacancies content of sheaf-like CeO_2_ and the catalytic activity of Pd/CeO_2_ catalyst in lean CH_4_ combustion was investigated by the means of X-ray powder diffraction (XRD), N_2_ physisorption, SEM, TEM, H_2_-temperature programmed reduction (H_2_-TPR), Raman spectroscopy and X-ray photoelectron spectroscopy (XPS). This work provides a new thought to the design and preparation of novel and efficient catalysts in catalytic combustion of lean methane. Moreover, the obtained catalyst can also be applied to the catalytic oxidation of other volatile organic compounds such as CO, formaldehyde and benzene. The other advantages of this study are that the preparation process of CeO_2_ is simple and easy for mass production—the obtained Pd/CeO_2_-SK catalyst can completely oxidize lean methane (1%) at 375 °C when Pd loading is only 1%. All of these are conducive to improving the economic benefits of its practical application.

## 2. Materials and Methods

### 2.1. Materials

The Ce(NO_3_)_3_·6H_2_O, polyvinyl pyrrolidone, urea, absolute ethanol, KOH, polyvinyl alcohol (PVA), and H_2_PdCl_4_ were supplied by Sinopharm Chemical Reagent Co.(Shanghai, China ), Ltd. All of the chemicals were used as received, and employed without further purification.

### 2.2. Preparation of Catalysts

As aforementioned, the hydrothermal method was employed to obtain the sheaf-like CeO_2_. In a typical preparation process, 10.0 mmol Ce(NO_3_)_3_·6H_2_O, 10 g polyvinyl pyrrolidone and 0.3 mol urea were dissolved in a mixture, which contains 50 mL of deionized water and 50 mL of absolute ethanol. This mixed solution was stirred for 0.5 h, and then put into an autoclave for 24 h at the temperature of 120 °C. After cooling, the precipitates were collected by filtration, and washed by deionized water and absolute ethanol more than once. Finally, the obtained precipitates were calcined for 4 h at the temperature of 500 °C after they were dried at 80 °C in air overnight. The obtained product was labeled as CeO_2_-S.

Then, 1.5 g dried CeO_2_-S prepared under the above conditions was added in 100 mL water. By adding KOH, the mixed solution had a final KOH mole concentration of about 4 mol/L and then the mixed solution was stirred for 40 min. Thereafter, the mixed solution was transferred into the autoclave, and then placed into a drying oven for 24 h at the temperature of 120 °C. Similarly, after cooling, the product was collected by filtration, washed by deionized water several times, dried at 80 °C in air overnight, and calcined at 500 °C for 4 h. The obtained product was marked as CeO_2_-SK.

Sheaf-like CeO_2_ supporting Pd catalysts were synthesized by a modified colloidal deposition method. Firstly, polyvinyl alcohol (PVA) was injected into 100 mg/L H_2_PdCl_4_ solution and sufficiently dissolved. Secondly, a certain amount of NaBH_4_ aqueous solution was added rapidly to obtain the colloidal palladium solution. Then, CeO_2_ powder was added into the above colloidal palladium solution and stirred for 24 h to achieve a designated content of 1 wt.% Pd on the CeO_2_ support. Finally, the obtained solid materials, viz., the Pd/CeO_2_-S and Pd/CeO_2_-SK, were collected by going through the identical procedure of filtration, washing, drying and calcination as compared with CeO_2_-S and CeO_2_-SK. According to a previous report, the presence of chlorides can affect the redox behavior of cerium oxide [28]. Therefore, in order to reduce the chlorides in the Pd/CeO_2_ catalysts, the washing times of the two Pd/CeO_2_ catalysts were kept the same during the washing process, and both of them were washed until the filtrate had no white precipitate after AgNO_3_ detection.

### 2.3. Catalytic Activity Measurements

The activity test for methane combustion over the catalysts was carried out in a quartz tube flow microreactor whose internal diameter is 6 mm. For each test, 200 mg of 40–60 mesh fresh catalyst was placed in the microreactor between two quartz glass wool layers. The reaction gas contained 1% CH_4_, 19% O_2_ and balanced Ar; and had a total flow rate of 100 mL/min, equivalent to a weight hourly space velocity (WHSV) of 30,000 mL/g∙h. The outlet gaseous mixture was analyzed online using a gas chromatograph (GC-2010, Shimadzu, Japan) to determine the component concentrations. The whole test process took about 2.5 h.

### 2.4. Catalyst Characterization

The catalysts surface area was analyzed by a TriStar 3000 Gas Absorption Analyzer (Micromeritics Instrument Co., Atlanta, GA, USA) and N_2_ physisorption at −195.8 °C. The samples were degassed at 200 °C and 6.7 Pa for 2 h prior to the measurement. The Pd content in the catalysts was determined by inductively coupled plasma atomic emission spectroscopy (ICP-AES; PerkinElmer Co., Waltham, MA, USA). X-ray powder diffraction (XRD) patterns of the catalysts were detected by an X-ray diffraction system (Bruker Corporation, Billerica, MA, USA) using Cu *Kα* radiation (154.06 pm, 40 kV and 40 mA). The diffraction spectra were collected over the 2*θ* range of 5–85° at a scanning rate of 4°/min.

The morphologies of the catalysts were analyzed at an operating voltage of 200 kV by a JSM-7001F scanning electron microscopy (SEM; JEOL Company, Tokyo, Japan). Meanwhile, characterization was also carried out by using both transmission electron microscopy (TEM; JEM-2010, JEOL Company, Tokyo, Japan) and high-resolution transmission electron microscopy (HRTEM; JEM-2010, JEOL Company, Tokyo, Japan) operating at 200 kV. To prepare the specimen for HRTEM, the catalyst sample was crushed to a fine powder and then a holey carbon film copper grid was dipped into the crushed powder.

Meanwhile, a Micromeritics AutoChem II 2920 Chemisorption Analyzer (Micromeritics Instrument Co., Atlanta, GA, USA) with a TCD detector was adopted for H_2_-temperature programmed reduction (H_2_-TPR). Typically, approximately 50 mg catalysts were reduced under a 10 vol % H_2_-Ar mixture. Before the H_2_-TPR analysis, the catalysts were treated under an air flow at 500 °C for 30 min, followed by purging with pure N_2_ at the same temperature for 30 min and then cooled down to 0 °C. After that, the reduction process took place in the temperature range of 0–900 °C.

In addition, Raman spectra were obtained using a Horiva Jobin Yvon LabRam HR800 Dual Microscope with a 514 nm Ar ion laser under ambient temperature. The X-ray photoelectron spectroscopy (XPS) analysis of the catalysts was performed on a spectrometer (ULVAC PHI-5800, Chanhassen, MN, USA) with the X-ray source of Al *Kα*. The standard binding energy of C 1*s* = 284.6 eV was used as the reference to shift the binding energies of the samples.

## 3. Results

### 3.1. XRD Results and Textural Properties

**Figure 1:**
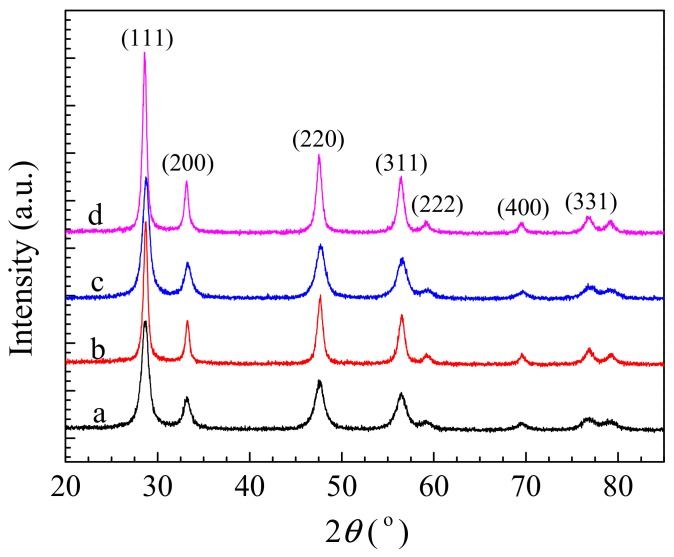
X-ray powder diffraction (XRD) patterns of (**a**) CeO_2_-S, (**b**) CeO_2_-SK, (**c**) Pd/CeO_2_-S and (**d**) Pd/CeO_2_-SK.

The XRD patterns of sheaf-like CeO_2_ supports and the Pd loaded catalysts are shown in Figure 1. Typical diffraction lines of ceria fluorite structure (JCPDS 34-0349) are observed for all of the samples. However, no distinct diffraction line for Pd species is detected in Figure 1, indicating that palladium species are finely dispersed on CeO_2_ surface and its size might be small.

Table 1 summarizes the Brunauer–Emmett–Teller surface area, average pore volume and pore size of sheaf-like CeO_2_ supports as well as the corresponding supported Pd catalysts. It showed that the surface area of CeO_2_-S support (66.5 m^2^/g) was very similar to that of CeO_2_-SK support (70.6 m^2^/g). After the deposition of Pd component, the surface areas of the obtained Pd catalysts remained almost unchanged as compare with that of the corresponding carriers. Meanwhile, as shown in Table 1, the Pd contents in both of the two Pd-containing catalysts were close to the designated value (1 wt.%).

### 3.2. SEM Results

**Figure 2:**
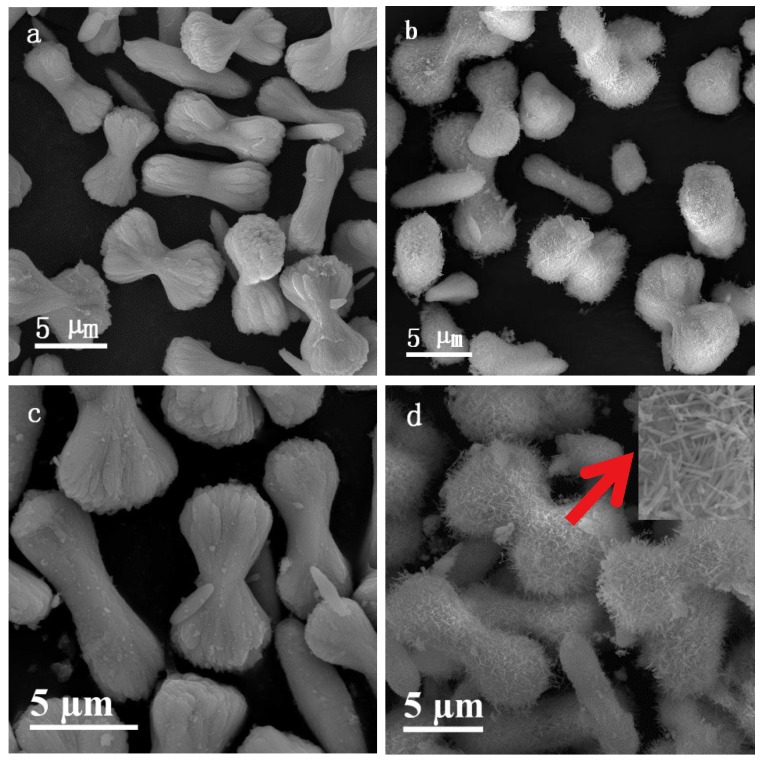
Scanning electron microscopy (SEM) images of (**a**) CeO_2_-S, (**b**) CeO_2_-SK, (**c**) Pd/CeO_2_-S and (**d**) Pd/CeO_2_-SK (the inset is a magnified SEM image).

SEM images of sheaf-like CeO_2_ supports as well as the Pd loaded catalysts are displayed in Figure 2. Clearly, the Pd loaded catalysts maintained the original morphology of those CeO_2_ carriers. The obtained CeO_2_-S support (Figure 2a) resembled a wheat bundle composed of many filamentous crystals, which was bandaged in its middle and fanning-out at both ends. Similar structures have been observed for CuO and Bi_2_S_3_ in the literature [29,30]. The average diameter of the individual nano-filament was about 10 nm and the bundles length was about 6–8 µm. Moreover, a smooth surface of the sheaves particles could be observed. However, after KOH etching, the surface of CeO_2_-SK bundled particles become rough due to the formation of a large number of nanorods (Figure 2c,d).

### 3.3. TEM and HRTEM Results

**Figure 3:**
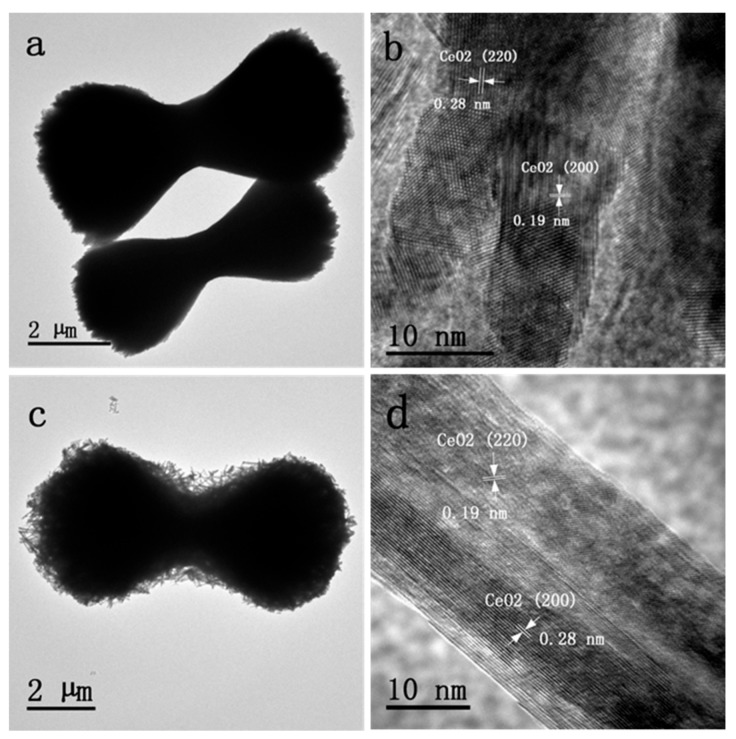
Transmission electron microscopy (TEM) and high-resolution transmission electron microscopy (HRTEM) images of (**a**,**b**) Pd/CeO_2_-S and (**c**,**d**) Pd/CeO_2_-SK.

Figure 3 presents the TEM and HRTEM images of supported Pd catalysts. It can be seen from Figure 3a,c that both Pd supported catalysts show a sheaf-like structure, and an abundance of nanorods were observed on the Pd/CeO_2_-SK surface; which was in accordance with the above SEM images. Moreover, as shown in Figure 3b, the complete structure of Pd/CeO_2_-S was too big, and therefore the HRTEM images were observed for the typical edge part of Pd/CeO_2_-S. The lattice plane spacing calculated from the HRTEM images was about 0.19 and 0.28 nm, which could be indexed as the (110) and (100) planes of CeO_2_, respectively. In the Pd/CeO_2_-SK HRTEM image (Figure 3d), the lattice fringes were clear and their spacing values were 0.19 and 0.28 nm, respectively; which were also attributed to (110) and (100) planes of CeO_2_. Although both Pd/CeO_2_-S and Pd/CeO_2_-SK catalysts exposed mainly the (110) and (100) crystal faces of CeO_2_, Pd/CeO_2_-SK had a larger amount of nanorods on its surface after KOH etching, and finally Pd/CeO_2_-SK owned more content of (110) and (100) CeO_2_ planes than Pd/CeO_2_-S. In addition, because the diffraction contrast of Pd and CeO_2_ was similar, no Pd particles were observed on both of the two Pd-containing catalysts [19].

### 3.4. H_2_-TPR Results

**Figure 4:**
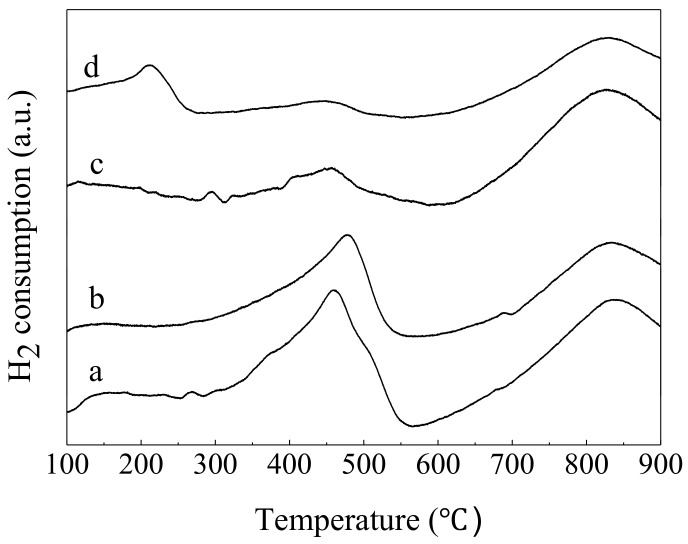
H_2_-temperature programmed reduction (H_2_-TPR) profiles of (**a**) CeO_2_-S, (**b**) CeO_2_-SK, (**c**) Pd/CeO_2_-S and (**d**) Pd/CeO_2_-SK.

The H_2_-TPR profiles of sheaf-like CeO_2_ supports and the Pd loaded catalysts are shown in Figure 4; the results of a quantitative analysis of the H_2_-TPR profiles are listed in Table 2. Obviously, the H_2_-TPR profiles of the two sheaf-like CeO_2_ supports are similar to each other. It can be seen that two main reduction peaks existed at 0–900 °C, and the peaks were believed to be related to the reduction of surface oxygen of CeO_2_ (at about 460 °C with a H_2_ uptake of 1170 μmol/g for CeO_2_-S and 985 μmol/g for CeO_2_-SK) and bulk CeO_2_ (at about 840 °C), respectively [16,31,32].

After loading the Pd component, the redox ability of CeO_2_ is changed. The CeO_2_ surface oxygen redox reduction peaks centered at 460 °C shift to 450 °C for both of Pd-containing catalysts, implying the promoted reducibility for Pd catalysts. Moreover, a new peak appears at around 210 °C for the Pd/CeO_2_-SK catalyst. It can be assigned to the reduction of PdO although its reduction temperature is higher compared with pure PdO [23]. As reported by Fu et al., this phenomenon could be mainly owing to the interaction between CeO_2_ and PdO [33]. Noticeably, the PdO species reduction peak is not observed in Pd/CeO_2_-S, indicating that Pd/CeO_2_-SK owns a much higher fraction of PdO species than that of Pd/CeO_2_-S.

SEM and TEM results show that both Pd/CeO_2_-S and Pd/CeO_2_-SK catalysts exhibit sheaf-like morphologies, but Pd/CeO_2_-SK possesses more nanorods enclosed mainly by CeO_2_ (100) and (110) facets. Previous studies reveal that the migration of lattice oxygen from bulk to surface on the (110) and (100) facet-dominated catalyst is much easier compared with that on the (111) facet-dominated one [16,34]. Therefore, the difference in their redox ability may be attributed to the exposure of larger amount of CeO_2_ (110) and (100) planes on Pd/CeO_2_-SK surface compared with Pd/CeO_2_-S.

### 3.5. Raman Spectroscopy

**Figure 5:**
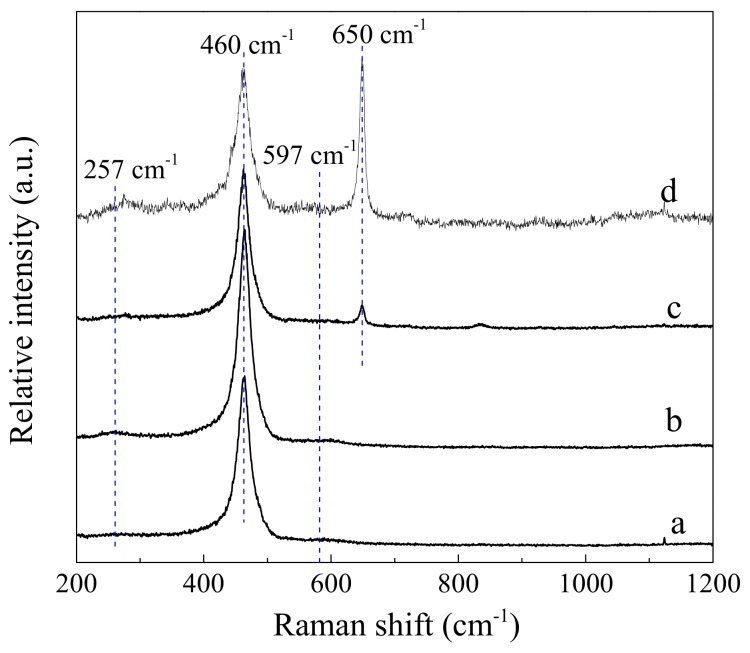
Raman spectra of (**a**) CeO_2_-S, (**b**) CeO_2_-SK, (**c**) Pd/CeO_2_-S and (**d**) Pd/CeO_2_-SK (its data is enlarged four times).

The Raman spectra of sheaf-like CeO_2_ supports and the Pd loaded catalysts are shown in Figure 5. For ease of observation, the Pd/CeO_2_-SK data is enlarged four times. For CeO_2_ supports, the main bonds at 460 cm^−1^ correspond to the F_2g_ Raman active mode of CeO_2_ fluorite structure [19,35,36,37]; the weak bands at about 597 cm^−1^ are assigned to the defect induced mode (D-mode), which should be associated with the existence of oxygen vacancies caused by Ce^3+^ ions in the CeO_2_ lattice [19,22,38]; the bands at 257 cm^−1^ are ascribed to the second order transverse acoustic mode (2TA-mode) [19].

After incorporating Pd component, the F_2g_ band of Pd catalysts is broader and slightly weaker compared with CeO_2_ supports, which is a sign of enhanced Pd–support interaction. Meanwhile, the surroundings environment of CeO_2_ surface is possibly changed by the introduction of Pd through an epitaxial contact between Pd and CeO_2_ supports. In addition, the observed widening and weakening of the F_2g_ band for the Pd-containing catalysts may be related to their lower crystallite size or the larger content of oxygen vacancies in ceria [39]. However, the Pd/CeO_2_ catalyst and the corresponding CeO_2_ support have a similar particle size as seen from the SEM images. Thus, the above changes of the CeO_2_ F_2g_ band in Pd-containing catalysts can be related to the larger fraction of oxygen vacancies on CeO_2_ surface. Noticeably, the Pd/CeO_2_-SK catalyst exhibited slightly intenser D-mode peak than Pd/CeO_2_-S, indicating that the former has a higher proportion of oxygen vacancies. Another obvious difference between the Raman spectra of supported Pd catalysts and the parent CeO_2_ carriers is a typical PdO band appeared at about 650 cm^−1^ on Pd catalysts, which can be ascribed to the B_1g_ mode of square planar [PdO_4_] subunits in PdO [19,40,41]. However, the intensity of this PdO band decreased greatly for Pd/CeO_2_-S catalyst, reflecting the lower content of PdO species on Pd/CeO_2_-S; which agreed well with the H_2_-TPR results. Furthermore, early studies showed that the reaction mechanism of CH_4_ combustion reaction over Pd-based catalysts usually follows the Mars–van Krevelen mechanism [42,43,44]. During the reaction, the decomposition and reformation of PdO species has been observed (PdO→Pd→PdO) [14]; that is, CH_4_ firstly reacts with O bounded with Pd to create CO_2_/H_2_O and oxygen vacancies, the generated oxygen vacancies are then supplemented by the gaseous oxygen or the bulk lattice oxygen. Obviously, the Pd oxides in Pd/CeO_2_ catalysts are very important for the lean CH_4_ combustion reaction. Misch et al. and Gholami et al. also discovered that PdO exhibits higher activity than that of metallic Pd in CH_4_ oxidation process [45,46]. Therefore, the higher content of PdO species on Pd/CeO_2_-SK may also imply higher activity compared with Pd/CeO_2_-S.

As reported in the literature, the degree of oxygen vacancies on CeO_2_ is related to the ratio of peak areas of D-mode to F_2g_ [22,39]. In this way, a higher D/F_2g_ ratio means a larger content of oxygen vacancies. The D/F_2g_ ratio of sheaf-like CeO_2_ carriers as well as the Pd loaded catalysts is presented in Table 3. It shows that the D/F_2g_ ratio of CeO_2_-SK support (0.104) and Pd/CeO_2_-SK catalyst (0.136) is much larger than that of CeO_2_-S (0.068) and Pd/CeO_2_-S (0.093), which reveals that the former samples own more amounts of oxygen vacancies. As mentioned above, after KOH etching, numerous nanorods are formed on the CeO_2_-SK surface, and these nanorods enclosed by CeO_2_ (100) and (110) facets. Sayle et al. demonstrated that the formation energy of oxygen vacancies on ceria planes is ranked in the descending order of (111), (100) and (110) [47], indicating that the anion vacancies are more easily formed on CeO_2_ (110) and (100) planes. As a result, the CeO_2_-SK support and Pd/CeO_2_-SK catalyst exhibit higher D/F_2g_ ratio, and a larger amount of oxygen vacancies are created on their surface.

### 3.6. XPS Results

**Figure 6:**
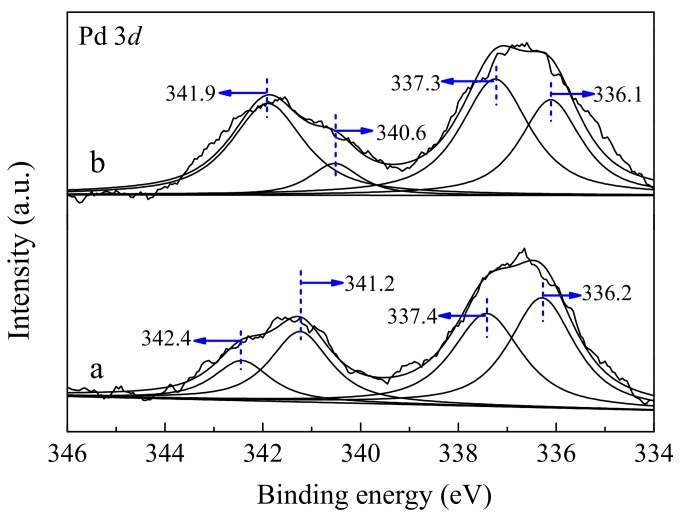
Pd 3*d* XPS spectra of (**a**) Pd/CeO_2_-S and (**b**) Pd/CeO_2_-SK.

The oxidation states and surface composition of the elements present in the as-made catalysts were analyzed by the XPS measurements. Figure 6 illustrates the Pd 3*d* XPS profiles of the two Pd loaded catalysts. Clearly, two Pd 3*d*_5/2_ peaks at 336.2, 337.4 eV and two Pd 3*d*_3/2_ peaks at 341.2 and 342.4 eV are observed for the Pd/CeO_2_-S catalyst, respectively. The peaks located at 336.2 and 341.2 eV can be attributed to the metallic Pd, and the peaks centered at 337.4 and 342.4 eV are related to the Pd oxides [22,48]. As for Pd/CeO_2_-SK catalyst, the Pd 3*d*_5/2_ peaks and 3*d*_3/2_ peaks shift to slightly lower binding energy. As shown in Table 3, approximately 50.0% of Pd species existed on the surface of Pd/CeO_2_-S in the form of metallic Pd; whereas, this value decreased to 45.8% for Pd/CeO_2_-SK. The results revealed that the concentration of PdO species on Pd/CeO_2_-SK surface was higher than that on Pd/CeO_2_-S, which was consistent with the surface content of PdO observed from the Raman analysis.

**Figure 7:**
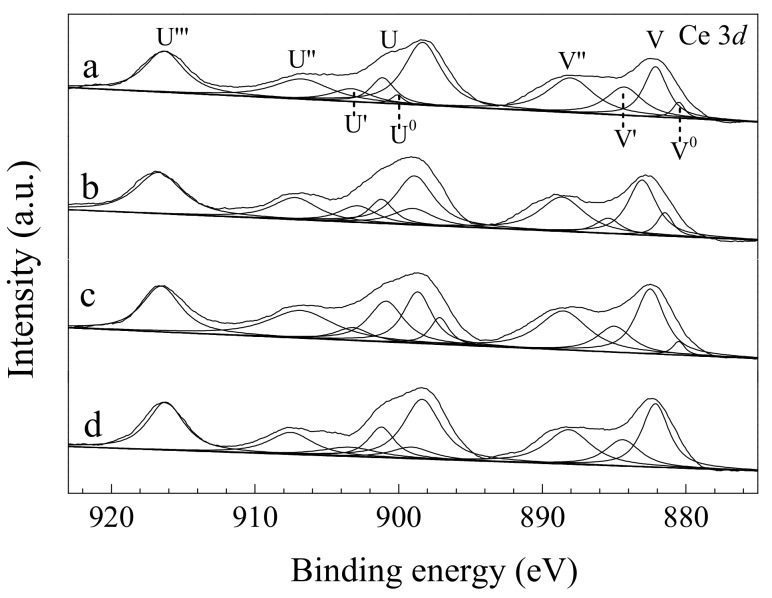
Ce 3*d* XPS spectra of (**a**) CeO_2_-S, (**b**) CeO_2_-SK, (**c**) Pd/CeO_2_-S and (**d**) Pd/CeO_2_-SK.

The Ce 3*d* XPS spectra of sheaf-like CeO_2_ supports and the Pd loaded catalysts are presented in Figure 7. It can be seen that the XPS spectra of CeO_2_ carriers and the Pd loaded catalysts exhibited ten peaks. The peaks labeled U and V belong to the spin-orbit components of Ce 3*d*_3/2_ and Ce 3*d*_5/2_, respectively [49,50]. In addition, the peaks marked by U, V, U’’, V’’, U’’’ and V’’’ are of Ce^4+^, while those denoted by U^0^, U’, V^0^ and V’ are Ce^3+^ characteristic peaks [51,52]. This reveals that two types of cerium oxides are present on the surface of all the samples, i.e., Ce^4+^ and Ce^3+^. It is generally believed that the surface Ce^3+^ results from the surface defects and surface oxygen vacancies, which have a positive impact on the lean methane combustion. Thus, the calculation of Ce^3+^ content is necessary for estimating the surface oxygen vacancies content. The area ratio [S1/(S1 + S2)] of the Ce^3+^ peaks area (S1) to the total area (S1 + S2, Ce^4+^ peaks area labels S2) of Ce 3d peaks is used to detect the surface Ce^3+^ content. Thus, a higher S1/(S1 + S2) ratio reflects a larger Ce^3+^ concentration. The calculated results are listed in Table 3. Clearly, CeO_2_-SK support (19.3) and Pd/CeO_2_-SK catalyst (21.0) exhibited higher Ce^3+^ content than those in CeO_2_-S (16.5) and Pd/CeO_2_-S (17.4), further confirming that more oxygen vacancies existed on the CeO_2_-SK support and Pd/CeO_2_-SK catalyst, coinciding with the Raman spectra results. Moreover, the loading of Pd also enhanced the Ce^3+^ concentration in the obtained Pd-containing catalysts. This phenomenon implies that the valence of elements of the CeO_2_ surface was changed owing to the interaction between Pd and CeO_2_.

**Figure 8:**
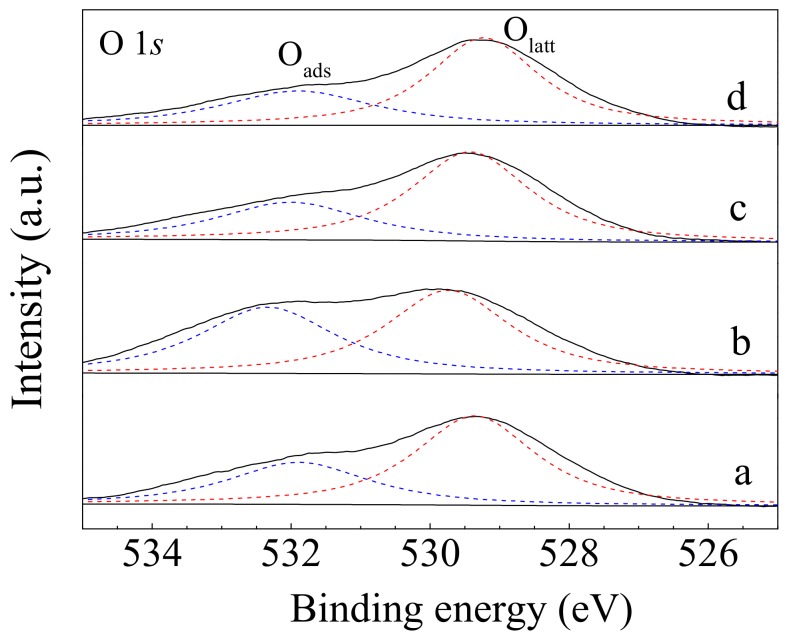
O 1*s* XPS spectra of (**a**) CeO_2_-S, (**b**) CeO_2_-SK, (**c**) Pd/CeO_2_-S and (**d**) Pd/CeO_2_-SK.

Figure 8 presents the O 1*s* XPS spectra of sheaf-like CeO_2_ supports and the Pd loaded catalysts. As seen from Figure 8, all of the samples have two typical peaks with different energies. The peak at 529.3–529.7 eV stands for the lattice oxygen species (O_latt_), while the other one at about 531.9–532.5 eV is related to the surface adsorbed oxygen (O_ads_) [49], which is usually considered active for oxidation reactions. As such, the ratio between the two kinds of oxygen species (O_ads_/O_latt_) was quantified based on the area of O_latt_ and O_ads_ (Table 2). The O_ads_/O_latt_ ratio was 0.55 for CeO_2_-S, whereas it dramatically increased to 0.97 for CeO_2_-SK; implying a greater tendency to form adsorbed oxygen species on the CeO_2_-SK surface. According to the above characterization results, after KOH etching, a large number of nanorods enclosed mainly by CeO_2_ (110) and (100) planes were produced on the CeO_2_-SK surface, which then promoted the creation of oxygen vacancies. These oxygen vacancies were easy to absorb oxygen to form active adsorbed oxygen species, and ultimately enhance the redox ability and catalytic performance of the obtained catalysts. In addition, it also could be seen from Table 3 that the addition of Pd caused a slight decrease of O_ads_/O_latt_ ratio, though Pd/CeO_2_-SK exhibit higher O_ads_/O_latt_ ratio than Pd/CeO_2_-S. This might be due to the fact that a few chemisorbed oxygen sites are occupied by Pd components.

### 3.7. CH_4_ Combustion Catalytic Activity

**Figure 9:**
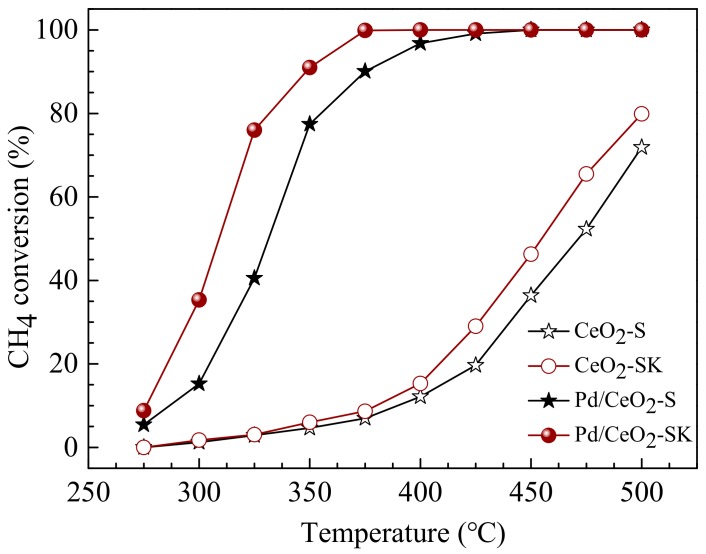
Methane conversion for the catalytic combustion over sheaf-like CeO_2_ supports and the corresponding supported Pd catalysts.

The catalytic activities of the sheaf-like CeO_2_ supports and the corresponding supported Pd catalysts in lean methane combustion are shown in Figure 9. In these tests, only CO_2_ and H_2_O as products were detected, indicating the good selectivity of the obtained catalysts in the complete oxidation of CH_4_.

It can be seen from Figure 9, CeO_2_-S and CeO_2_-SK supports show poor activity in lean methane combustion and the CH_4_ conversion values at 500 °C are only 71.9% and 79.9%, respectively. After deposition Pd component, the CH_4_ conversion of the Pd-containing catalysts was increased significantly, suggesting the important role of Pd in lean methane combustion. Moreover, it also shows that the activity of the as prepared Pd catalysts depended largely on the structural feature of CeO_2_ supports such as its exposing crystal face; the Pd/CeO_2_-SK catalyst shows a high activity, superior to the Pd/CeO_2_-S catalyst. Over the Pd/CeO_2_-SK catalyst, CH_4_ conversion reached 91% at 350 °C and 100% at 375 °C, while the full conversion of CH_4_ was achieved at 425 °C for Pd/CeO_2_-S catalyst.

**Figure 10:**
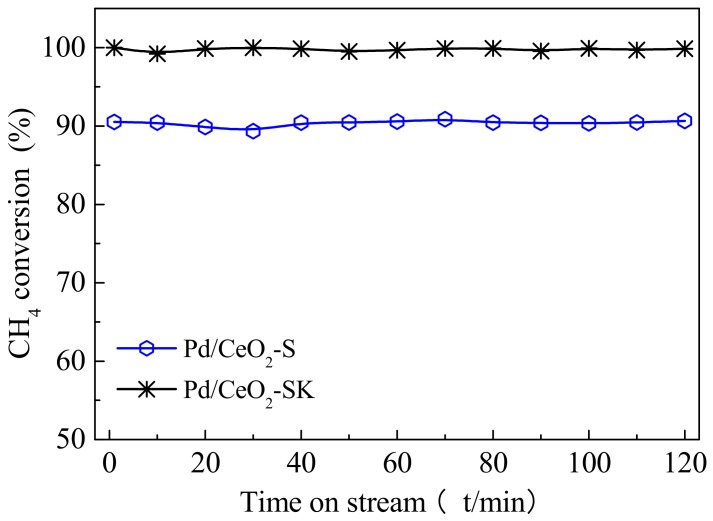
Long-term stability test for CH_4_ catalytic combustion over Pd/CeO_2_-S and Pd/CeO_2_-SK catalysts at 375 °C.

Long-term tests were also performed for CH_4_ catalytic combustion over the Pd/CeO_2_-S and Pd/CeO_2_-SK catalysts at 375 °C, as shown in Figure 10. Clearly, the Pd/CeO_2_-S catalyst displays a CH_4_ conversion about 90% and that of the Pd/CeO_2_-SK catalyst is approximately 100% during the 120 min test process. Both of the two Pd-containing catalysts show good thermal stability. Moreover, as shown in Figures S1 and S2 in the Supplementary Information, there are no structural changes for the Pd/CeO_2_-S and Pd/CeO_2_-SK catalysts after the long-term tests, in comparison with the fresh ones.

The above characterization results can explain the outstandingly activity of Pd/CeO_2_-SK catalyst in the lean methane combustion. According to the ICP, N_2_ physisorption and SEM results, the Pd supported catalysts were very similar in their Pd loading, surface area and morphologies. SEM and TEM results show that Pd/CeO_2_-SK surface had numerous nanorods enclosed by CeO_2_ (100) and (110) facets after KOH etching, which is beneficial to the formation of oxygen vacancies. These oxygen vacancies help absorb oxygen to form active adsorbed oxygen species and ultimately improve the reduction ability and catalytic performance of Pd/CeO_2_-SK. Moreover, as seen from the H_2_-TPR and Raman spectra results, the Pd/CeO_2_-SK catalyst possessed higher content of catalytic active PdO species than that of Pd/CeO_2_-S. As a result, the excellent performance of Pd/CeO_2_-SK in lean methane combustion was expected.

## 4. Conclusions

Sheaf-like CeO_2_ in microscale was prepared by the hydrothermal method. The as-made sheaf-like CeO_2_ was then further etched with KOH for obtaining an imperfect fluorite structure with higher content of oxygen vacancies as well as improved oxygen mobility. With the as-made and etched sheaf-like CeO_2_ as supports respectively, two Pd/CeO_2_ catalysts were obtained for lean methane combustion by a modified colloidal deposition method.

After KOH etching, CeO_2_-SK surface shows numerous nanorods enclosed by CeO_2_ (100) and (110) facets and Pd/CeO_2_-SK exhibited much higher activity compares with Pd/CeO_2_-S; over the Pd/CeO_2_-SK catalyst, CH_4_ conversion reached 100% at 375 °C, while the full conversion of CH_4_ was achieved at 425 °C for Pd/CeO_2_-S catalyst. The larger proportion of CeO_2_ (100) and (110) planes in Pd/CeO_2_-SK enhance the creation of oxygen vacancies and oxygen migration, which had a positive impact on the activity of lean methane combustion. Moreover, Pd/CeO_2_-SK catalyst had higher content of catalytic active PdO species compares with Pd/CeO_2_-S catalyst due to the interaction between the CeO_2_ (100) and (110) planes and palladium species. All these contribute to the excellent activity of Pd/CeO_2_-SK in lean methane combustion.

## Figures and Tables

**Table 1:** Textural properties and Pd loading of sheaf-like CeO_2_ supports and the Pd loaded catalysts.

Samples	Pd Loading (wt.%)	S_BET_ (m^2^/g)	Average Pore Size (nm)	Pore Volume (cm^3^/g)
CeO_2_-S	-	66.5	4.2	0.0626
CeO_2_-SK	-	70.6	5.0	0.0654
Pd/CeO_2_-S	0.94	65.6	4.1	0.0602
Pd/CeO_2_-SK	0.93	68.7	4.6	0.0636

**Table 2:** Quantitative analysis of the H_2_-TPR profiles of sheaf-like CeO_2_ supports and the Pd loaded catalysts.

Samples	Peak Position (°C)	H_2_ Uptake (μmol/g)	Theoretical H_2_ Uptake (µmol/g) ^a^
CeO_2_-S	460	1170	2904
CeO_2_-SK	466	985	2904
Pd/CeO_2_-S	450	448	2954
Pd/CeO_2_-SK	210; 450	110; 332	2954

^a^ Theoretical H_2 _uptake was determined as the quantity of H_2_ required for the reduction of Pd catalysts and corresponding CeO_2_ supports by assuming that CeO_2_ and PdO are stoichiometrically reduced to Ce_2_O_3_ and Pd, respectively.

**Table 3:** Raman spectra and X-ray photoelectron spectroscopy (XPS) results of sheaf-like CeO_2_ supports and the Pd loaded catalysts.

Samples	D/F_2g_	Pd^0^ Content (%)	Ce^3+^ Content (%)	O_ads_/O_latt_ Ratio
CeO_2_-S	0.068	-	16.5	0.55
CeO_2_-SK	0.104	-	19.3	0.97
Pd/CeO_2_-S	0.093	50.0	17.4	0.48
Pd/CeO_2_-SK	0.136	45.8	21.0	0.63

## Supplementary Materials

The following are available online at https://www.mdpi.com/2079-4991/10/1/31/s1: Figure S1: XRD patterns of the Pd/CeO_2_-S and Pd/CeO_2_-SK catalysts before and after the long-term test in lean methane combustion; Figure S2: SEM images of the Pd/CeO_2_-S and Pd/CeO_2_-SK catalysts after the long-term test in lean methane combustion.

## References

[1] Liu F.X., Sang Y.Y., Ma H.W., Li Z.P., Gao Z.M. (2017). Nickel oxide as an effective catalyst for catalytic combustion of methane. J. Nat. Gas Sci. Eng..

[2] Zhou F.B., Xia T.Q., Wang X.X., Zhang Y.F., Sun Y.N., Liu J.S. (2016). Recent developments of coal mine methane extraction and utilization in China: A review. J. Nat. Gas Sci. Eng..

[3] Guo T., Du J., Wu J., Wang S., Li J. (2016). Structure and kinetic investigations of surfacestepped CeO_2_-supported Pd catalysts for low-concentration methane oxidation. Chem. Eng. J..

[4] Zhang H., Li P., Hui N., Liang J., Ding Y., Liu T. (2017). The microstructure and methane catalytic combustion of ceria composite materials modified with tourmaline particles. J. Alloys Compd..

[5] Zhang Y., Qin Z., Wang G., Zhu H., Dong M., Li S., Wu Z., Li Z., Wu Z., Zhang J. (2013). Catalytic performance of MnO*_x_*-NiO composite oxide in lean methane combustion at low temperature. Appl. Catal. B Environ..

[6] Ercolino G., Stelmachowski P., Kotarba A., Specchia S. (2017). Reactivity of mixed iron-cobalt spinels in the lean methane combustion. Top. Catal..

[7] Wang B., Qin Z., Wang G., Wu Z., Fan W., Zhu H., Li S., Zhang Y., Li Z., Wang J. (2017). Catalytic combustion of lean methane at low temperature over palladium on a CoO*_x_*-SiO_2_ composite support. Catal. Lett..

[8] Ercolino G., Karimi S., Stelmachowski P., Specchia S. (2017). Catalytic combustion of residual methane on alumina monoliths and open cell foams coated with Pd/Co_3_O_4_. Chem. Eng. J..

[9] Guo T., Du J., Li J. (2016). The effects of ceria morphology on the properties of Pd/ceria catalyst for catalytic oxidation of low-concentration methane. J. Mater. Sci..

[10] Schwartz W.R., Pfefferle L.D. (2012). Combustion of methane over palladium-based catalysts: support interactions. J. Phys. Chem. C.

[11] Fujimoto K., Ribeiro F.H., Avalos-Borja M., Iglesia E. (1998). Structure and reactivity of PdO*_x_*/ZrO_2_ catalysts for methane oxidation at low temperatures. J. Catal..

[12] Xin Y., Lieb S., Wang H., Law C.K. (2013). Kinetics of catalytic oxidation of methane over palladium oxide by wire microcalorimetry. J. Phys. Chem. C.

[13] Chenakin S.P., Melaet G., Szukiewicz R., Kruse N. (2014). XPS study of the surface chemical state of a Pd/(SiO_2_+TiO_2_) catalyst after methane oxidation and SO_2_ treatment. J. Catal..

[14] Farrauto R.J., Lampert J.K., Hobson M.C., Waterman E.M. (1995). Thermal decomposition and reformation of PdO catalysts; support effects. Appl. Catal. B Environ..

[15] Zhang R., Lu K., Zong L., Tong S., Wang X., Feng G. (2017). Gold supported on ceria nanotubes for CO oxidation. Appl. Surf. Sci..

[16] Li S., Zhu H., Qin Z., Wang G., Zhang Y., Wu Z., Li Z., Chen G., Wu Z., Zheng L. (2014). Morphologic effects of nano CeO_2_-TiO_2_ on the performance of Au/CeO_2_-TiO_2_ catalysts in low-temperature CO oxidation. Appl. Catal. B Environ..

[17] Sudarsanam P., Mallesham B., Reddy P.S., Großmann D., Grünert W., Reddy B.M. (2014). Nano-Au/CeO_2_ catalysts for CO oxidation: Influence of dopants (Fe, Laand Zr) on the physicochemical properties and catalytic activity. Appl. Catal. B Environ..

[18] Cordatos H., Bunluesin T., Stubenrauch J., Vohs J.M., Gorte R.J. (1996). Effect of ceria structure on oxygen migration for Rh/ceria catalysts. J. Phys. Chem..

[19] Ma J., Lou Y., Cai Y., Zhao Z., Wang L., Zhan W., Guo Y.L., Guo Y. (2018). The relationship between the chemical state of Pd species and the catalytic activity for methane combustion on Pd/CeO_2_. Catal. Sci. Technol..

[20] Dai Q., Bai S., Lou Y., Wang X., Guo Y., Lu G. (2016). Sandwich-like PdO/CeO_2_ nanosheet@HZSM-5 membrane hybrid composite for methane combustion: Self-redispersion, sintering-resistance and oxygen, water-tolerance. Nanoscale.

[21] Mayernick A.D., Janik M.J. (2011). Methane oxidation on Pd-Ceria: A DFT study of the mechanism over Pd*_x_*Ce_1-*x*_O_2_, Pd, and PdO. J. Catal..

[22] Tan H., Wang J., Yu S., Zhou K. (2015). Support morphology-dependent catalytic activity of Pd/CeO_2_ for formaldehyde oxidation. Environ. Sci. Technol..

[23] Lei Y., Li W., Liu Q., Lin Q., Zheng X., Huang Q., Guan S., Wang X., Wang C., Li F. (2018). Typical crystal face effects of different morphology ceria on the activity of Pd/CeO_2_ catalysts for lean methane combustion. Fuel.

[24] Guo H., He Y., Wang Y., Liu L., Yang X., Wang S., Huang Z., Wei Q. (2013). Morphology-controlled synthesis of cage-bell Pd@CeO_2_ structured nanoparticle aggregates as catalysts for the low-temperature oxidation of CO. J. Mater. Chem. A.

[25] Guo T.Y., Du J.P., Wu J.T., Li J.P. (2016). Palladium catalyst supported on stair-like microstructural CeO_2_ provides enhanced activity and stability for low-concentration methane oxidation. Appl. Cata. A General..

[26] Comotti M., Li W.C., Spliethoff B., Schüth F. (2006). Support effect in high activity gold catalysts for CO oxidation. J. Am. Chem. Soc..

[27] Wang G., Li W., Jia K., Spliethoff B., Schüth F., Lu A. (2009). Shape and size controlled α-Fe_2_O_3_ nanoparticles as supports for gold-catalysts: Synthesis and influence of support shape and size on catalytic performance. Appl. Catal. B Environ..

[28] Bernal S., Calvino J.J., Cifredo G.A., Gatica J.M., Pérez Omil J.A., Laachir A., Perrichon V. (1995). Influence of the nature of the metal precursor salt on the redox behaviour of ceria in Rh/CeO_2_ catalysts. Surf. Sci. Catal..

[29] Zhang M., Xu X.D., Zhang M.L. (2008). Hydrothermal synthesis of sheaf-like CuO via ionic liquids. Mater. Lett..

[30] Tang J., Paul Alivisatos A. (2006). Crystal splitting in the growth of Bi_2_S_3_. Nano Lett..

[31] Zhu H., Qin Z., Shan W., Shen W., Wang J. (2004). Pd/CeO_2_-TiO_2_ catalyst for CO oxidation at low temperature: a TPR study with H_2_ and CO as reducing agents. J. Catal..

[32] Hu F., Chen J., Peng Y., Song H., Li K., Li J. (2018). Novel nanowire self-assembled hierarchical CeO_2_ microspheres for low temperature toluene catalytic combustion. Chem. Eng. J..

[33] Fu Q., Wagner T. (2007). Interaction of nanostructured metal overlayers with oxide surfaces. Surf. Sci. Rep..

[34] Mai H.X., Sun L.D., Zhang Y.W., Si R., Feng W., Zhang H.P., Liu H.C., Yan C.H. (2005). Shape-selective synthesis and oxygen storage behavior of ceria nanopolyhedra, nanorods, and nanocubes. J. Phys. Chem. B.

[35] Li S., Zhang Y., Li X., Yang X., Li Z., Wang R., Zhu H. (2018). Preferential oxidation of CO in H_2_-rich stream over Au/CeO_2_-NiO catalysts: effect of the preparation method. Catal. Lett..

[36] Francisco M.S.P., Mastelaro V., Nascente P.A.P., Florentino A. (2001). Activity and characterization by XPS, HR-TEM, Raman spectroscopy, and bet surface area of CuO/CeO_2_-TiO_2_ catalysts. J. Phys. Chem. B.

[37] Peng C., Lia H., Liaw B., Chen Y. (2011). Removal of CO in excess hydrogen over CuO/Ce_1−*x*_Mn*_x_*O_2_ catalysts. Chem. Eng. J..

[38] Reddy B.M., Khan A., Yamada Y., Kobayashi T., Loridant S., Volta J. (2003). Structural characterization of CeO_2_-MO_2_ (M = Si^4+^, Ti^4+^ and Zr^4+^) mixed oxides by Raman spectroscopy, X-ray photoelectron spectroscopy, and other techniques. J. Phys. Chem. B.

[39] Hernández W.Y., Centeno M.A., Romero-Sarria F., Odriozola J.A. (2009). Synthesis and characterization of Ce_1-*x*_Eu*_x_*O_2-*x*/2_ mixed oxides and their catalytic activities for CO oxidation. J. Phys. Chem. C.

[40] Demoulin O., Navez M., Gaigneaux E.M., Ruiz P., Mamede A.S., Granger P., Payen E. (2003). Operando resonance Raman spectroscopic characterisation of the oxidation state of palladium in Pd/γ-Al_2_O_3_ catalysts during the combustion of methane. Phys. Chem. Chem. Phys..

[41] McBride J.R., Hass K.C., Weber W.H. (1991). Resonance-Raman and lattice-dynamics studies of single-crystal PdO. Phys. Rev. B.

[42] Wu Z., Deng J., Liu Y., Xie S., Jiang Y., Zhao X., Yang J., Arandiyan H., Guo G., Dai H. (2015). Three-dimensionally ordered mesoporous Co_3_O_4_-supported Au-Pd alloy nanoparticles: High-performance catalysts for methane combustion. J. Catal..

[43] Lou Y., Ma J., Hu W., Dai Q., Wang L., Zhan W., Guo Y., Cao X.M., Guo Y., Hu P. (2016). Low-temperature methane combustion over Pd/H-ZSM-5: active Pd sites with specific electronic properties modulated by acidic sites of H-ZSM-5. ACS Catal..

[44] Specchia S., Conti F., Specchia V. (2010). Kinetic studies on Pd/Ce*_x_*Zr_1-*x*_O_2_ Catalyst for methane combustion. Ind. Eng. Chem. Res..

[45] Misch L.M., Kurzman J.A., Derk A.R., Kim Y., Seshadri R., Metiu H., McFarland E.W., Stucky G.D. (2011). C-H bond activation by Pd-substituted CeO_2_: substituted ions versus reduced species. Chem. Mater..

[46] Gholami R., Smith K.J. (2015). Activity of PdO/SiO_2_ catalysts for CH_4_ oxidation following thermal treatments. Appl. Catal. B Environ..

[47] Sayle T.X.T., Parker S.C., Sayle D.C. (2005). Oxidising CO to CO_2_ using ceria nanoparticles. Phys. Chem. Chem. Phys..

[48] Qian K., Huang W.X. (2011). Au–Pd alloying-promoted thermal decomposition of PdO supported on SiO_2_ and its effect on the catalytic performance in CO oxidation. Catal. Today.

[49] Yang S., Zhou F., Liu Y., Zhang L., Chen Y., Wang H., Tian Y., Zhang C., Liu D. (2019). Morphology effect of ceria on the performance of CuO/CeO_2_ catalysts for hydrogen production by methanol steam reforming. Int. J. Hydrogen Energy.

[50] Scirè S., Crisafulli C., Riccobene P.M., Patanè G., Pistone A. (2012). Selective oxidation of CO in H_2_-rich stream over Au/CeO_2_ and Cu/CeO_2_ catalysts: An insight on the effect of preparation method and catalyst pretreatment. Appl. Catal. A Gen..

[51] Wang B., Chi C., Xu M., Wang C., Meng D. (2017). Plasma-catalytic removal of toluene over CeO_2_-MnO*_x_* catalysts in an atmosphere dielectric barrier discharge. Chem. Eng. J..

[52] Bêche E., Charvin P., Perarnau D., Abanades S., Flamant G. (2008). Ce 3d XPS investigation of cerium oxides and mixed cerium oxide (Ce_x_Ti_y_O_z_). Surf. Interface Anal..

