# Supplementary material for: Catalytic Performance of Palladium Supported on Sheaf-Like Ceria in the Lean Methane Combustion
Source: Nanomaterials (Basel). 2019 Dec 21;10(1):31. doi: 10.3390/nano10010031 (PMC7022713; doi:10.3390/nano10010031)
Supplement: Supplementary file 1 [file nanomaterials-10-00031-s001.pdf]

Supplementary materials

Catalytic Performance of Palladium Supported on Sheaf-Like Ceria in the Lean Methane Combustion

Shuna Li [1,*], Yagang Zhang [2], Jing Shi [3,*], Gang Zhu [1], Yanxiang Xie [1], Zhikai Li [4], Ruiyi Wang [4] and Huaqing Zhu [4,*]

1. Xi'an Key Laboratory on Intelligent Additive Manufacturing Technologies, The Key Laboratory for Surface Engineering and Remanufacturing in Shaanxi Province, School of Chemical Engineering, Xi'an University, Xi'an 710065, Shaanxi, China; zg_503a@163.com (G.Z.); yxxie@163.com (Y.X.)
2. Department of Chemistry and Chemical Engineering, Xi'an University of Science and Technology, Xi'an 710054, Shaanxi, China; zhangyg04@126.com
3. Department of Mechanical and Materials Engineering, University of Cincinnati, Cincinnati, OH 45221, USA
4. State Key Laboratory of Coal Conversion, Institute of Coal Chemistry, Chinese Academy of Sciences, P.O. Box 165, Taiyuan 030001, Shanxi, China; lizhikai@sxicc.ac.cn (Z.L.); wangruiyi@sxicc.ac.cn (R.W.)

* Correspondence: lishuna165@126.com (S.L.); jing.shi@uc.edu (J.S.); zhhq@sxicc.ac.cn (H.Z.); Tel.: +86-029-88269385 (S.L.)

The supplementary materials include more characterization results.

More characterization results

Figure S1 and Figure S2 show the XRD patterns and SEM images of the Pd/CeO_2-S and Pd/CeO_2-SK catalysts after the long-term tests. It can be seen that the XRD patterns and SEM images of Pd/CeO_2-S and Pd/CeO_2-SK catalysts remain almost unchanged in comparison with the fresh catalysts, indicating that there are no structural changes for Pd/CeO_2-S and Pd/CeO_2-SK catalysts after the long-term tests, in comparison with the fresh ones.

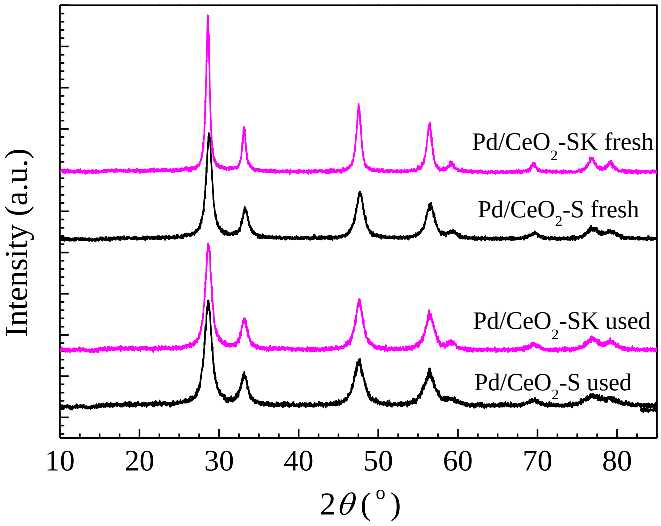

Figure S1. XRD patterns of the Pd/CeO_2-S and Pd/CeO_2-SK catalysts after the long-term test in lean methane combustion.

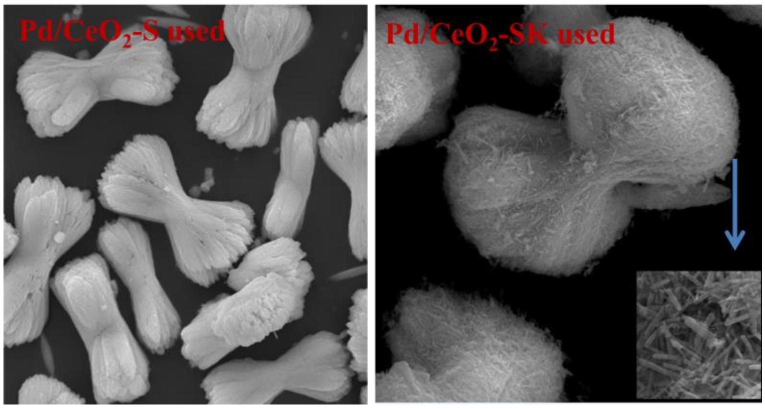

Figure S2. SEM images of the Pd/CeO_2-S and Pd/CeO_2-SK catalysts after the long-term test in lean methane combustion.
